# Dyadic Data on U.S. Chinese Older Adults and Their Adult Children: Study Design and Sample Overview

**DOI:** 10.1093/geroni/igab046.765

**Published:** 2021-12-17

**Authors:** Dexia Kong, XinQi Dong

**Affiliations:** 1 The Chinese University of Hong Kong, Hong Kong , Hong Kong; 2 Rutgers University, Rutgers Institute for Health, New Jersey, United States

## Abstract

This paper aims to describe study design of the unique dyadic older Chinese American-adult children dataset, and present sample characteristics of the dyads. A total of 807 older parents were matched with their adult children (characteristics of matched versus not matched participants will be compared). On average, adult children were 48 years old, had 12 years of education, lived with 3 persons in household, had 2 children, and lived in U.S. for 17 years. Approximately 65% of the adult children sample were female, 82% married, 93% preferred to speak Chinese dialects, and over 97% foreign-born immigrants. On the other hand, older parents were 74 years old, had 7 years of education, lived with 3 persons in household, had 3 children, and lived in U.S. for 17 years on average. About 60% of the older parent sample were female, 73% married, over 99% foreign-born immigrants who preferred to speak Chinese dialects.

